# A Curcumin Derivative Activates TFEB and Protects Against Parkinsonian Neurotoxicity in Vitro

**DOI:** 10.3390/ijms21041515

**Published:** 2020-02-22

**Authors:** Ziying Wang, Chuanbin Yang, Jia Liu, Benjamin Chun-Kit Tong, Zhou Zhu, Sandeep Malampati, Sravan Gopalkrishnashetty Sreenivasmurthy, King-Ho Cheung, Ashok Iyaswamy, Chengfu Su, Jiahong Lu, Juxian Song, Min Li

**Affiliations:** 1 Mr. & Mrs. Ko Chi-Ming Centre for Parkinson’s Disease Research, School of Chinese Medicine, Hong Kong Baptist University, Hong Kong SAR 000000, China; wangziying.12@163.com (Z.W.); nkyangchb@gmail.com (C.Y.); liujiatheone@hotmail.com (J.L.); benjamintck@gmail.com (B.C.-K.T.); zzhou1022@gmail.com (Z.Z.); deepu.pharma08@gmail.com (S.M.); sravangs@gmail.com (S.G.S.); kingho@hkbu.edu.hk (K.-H.C.); ashokenviro@gmail.com (A.I.); suchengfu@hkbu.edu.hk (C.S.); 2 Institute for Research and Continuing Education, Hong Kong Baptist University, Shenzhen 518000, China; 3 State Key Laboratory of Quality Research in Chinese Medicine, Institute of Chinese Medical Sciences, University of Macau, Macau SAR 000000, China; jiahonglu@um.edu.mo; 4 Medical College of Acupuncture-Moxibustion and Rehabilitation, Guangzhou University of Chinese Medicine, Guangzhou 510000, China

**Keywords:** TFEB, Parkinson’s disease, α-synuclein, curcumin derivatives, MTORC1

## Abstract

TFEB (transcription factor EB), which is a master regulator of autophagy and lysosome biogenesis, is considered to be a new therapeutic target for Parkinson’s disease (PD). However, only several small-molecule TFEB activators have been discovered and their neuroprotective effects in PD are unclear. In this study, a curcumin derivative, named E4, was identified as a potent TFEB activator. Compound E4 promoted the translocation of TFEB from cytoplasm into nucleus, accompanied by enhanced autophagy and lysosomal biogenesis. Moreover, TFEB knockdown effectively attenuated E4-induced autophagy and lysosomal biogenesis. Mechanistically, E4-induced TFEB activation is mainly through AKT-MTORC1 inhibition. In the PD cell models, E4 promoted the degradation of α-synuclein and protected against the cytotoxicity of MPP^+^ (1-methyl-4-phenylpyridinium ion) in neuronal cells. Overall, the TFEB activator E4 deserves further study in animal models of neurodegenerative diseases, including PD.

## 1. Introduction

Parkinson’s disease (PD) is the second most common neurodegenerative disease in the world, affecting around 6.2 million people in 2015 [1]. The major hallmarks of PD are the loss of dopaminergic neurons (DAs) in the substantia nigra pars compacta (SNpc) and abnormal deposition of α-synuclein (α-syn) in different brain regions [2]. Unfortunately, the current drugs for PD only reduce motor symptoms while producing severe side effects [3,4]. Thus, neuroprotective or disease-modifying drugs that can slow down or stop the disease progression are urgently needed.

Accumulating evidence has demonstrated that the impairment of the autophagy-lysosome pathway (ALP) plays an important role in the pathogenesis of PD. Therefore, stimulating ALP represents a promising strategy for PD treatment [5,6,7,8]. ALP, a cellular process for degrading misfolded cellular proteins and damaged organelles in lysosomes [6], has been proved to be tightly controlled by the transcription factor EB (TFEB) [9]. Several recent studies have investigated the roles and therapeutic potential of TFEB in PD [10,11,12,13,14]. Defective ALP and decreased TFEB activity was found in postmortem PD midbrains [10]. The overexpression of TFEB afforded protection against both α-syn [10] and MPTP (1-methyl-4-phenyl-1,2,3,6-tetrahydropyridine) toxicity [11] and improved motor function in the PD animal models. However, small-molecule activators of TFEB with good brain bioavailability should be discovered and evaluated in PD animal models.

Previously, we have identified several TFEB activators from curcumin derivatives [15], one of which directly binds to and activates TFEB without inhibiting the MTORC1 pathway and it exerts neuroprotective effects in animal models of Alzheimer’s disease [16]. In this study, we characterized another curcumin derivative, termed E4, as an MTORC1-dependent activator of TFEB. We found that compound E4 potently activated TFEB via the AKT-MTORC1 pathway, promoted autophagy flux and lysosomal biogenesis, and protected against α-syn and MPTP toxicity in vitro.

## 2. Results

### 2.1. Compound E4 Promoted the Nuclear Translocation of Endogenous and Exogenous TFEB in Vitro

**Figure 1:**
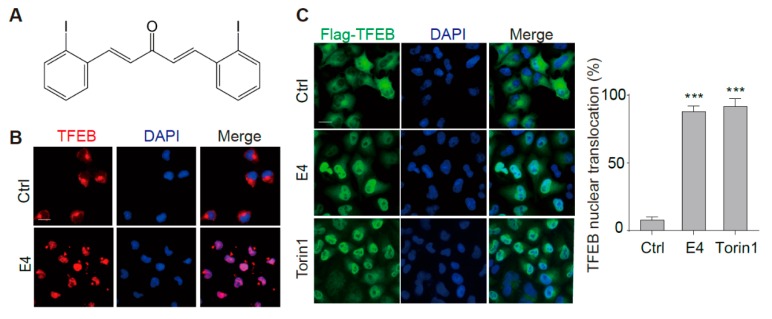

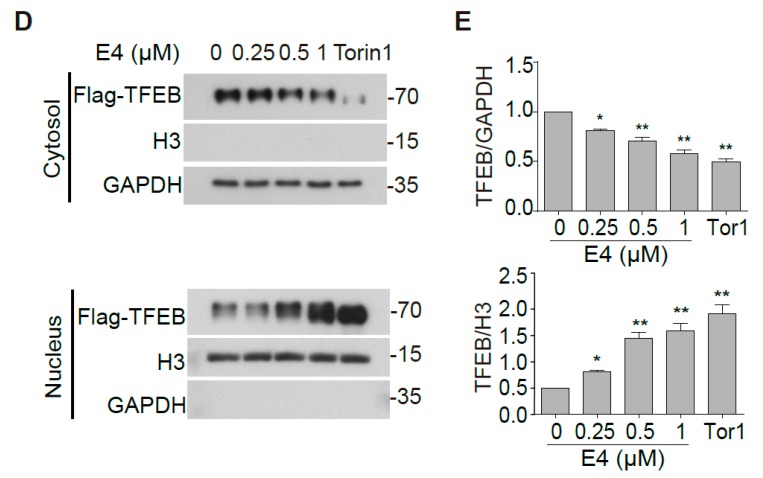
Compound E4 promoted the nuclear translocation of endogenous and exogenous transcription factor EB (TFEB) in vitro. (**A**) Chemical structure of E4. (**B**) E4 significantly promoted the nuclear intensity of endogenous TFEB in N2a cells compared to control (0.1% DMSO). After being treated with E4 (1 μM) for 24 h, N2a cells were fixed and stained with the anti-TFEB antibody (red) and DAPI (blue). Representative images are shown. Scale bar: 15 μm. (**C**) E4 significantly promoted the nuclear intensity of TFEB in CF-7 cells compared to control (0.1% DMSO). After being treated with E4 (1 μM) and positive control Torin1 (250 nM) for 24 h, HeLa cells stably expressing 3XFlag-TFEB (CF-7) were fixed and stained with the anti-Flag antibody (green) and DAPI (blue). Representative images are shown. Scale bar: 15 μm. Quantification of the number of cells with nuclear TFEB localization. Data are presented as mean ± SEM of three replicates in a representative experiment. At least 100 cells were analyzed in each treatment group. (**D**) The expression of Flag-TFEB in the cytosol and the nucleus in CF-7 cells were detected by Western blotting after being treated with indicated compounds for 6 h. (**E**) Relative intensity of TFEB is normalized to that of GAPDH and H3. Data are presented as mean ± SEM of three replicates in a representative experiment. * *p* < 0.05, ** *p* < 0.01, *** *p* < 0.001.

In normal conditions, TFEB locates in the cytoplasm. Being stimulated by various stresses, TFEB is de-phosphorylated and transported into the nucleus, where it binds to and activates multiple genes controlling autophagy and lysosomal biogenesis [17]. We monitored the localization of TFEB by immunostaining to determine whether compound E4 (Figure 1A) activates TFEB. E4 significantly promoted the nuclear intensity of endogenous TFEB in N2a cells (Figure 1B), and the stably overexpressed TFEB in HeLa cells (CF7) (Figure 1C). The TFEB activator Torin 1 was used as a positive control [18]. Furthermore, the result from Western blots of cytosolic/nuclear fractions confirmed that E4 activated TFEB (Figure 1D,E). No cytotoxicity was observed for E4 at the tested concentrations (Figure S1).

### 2.2. Compound E4 Promoted Autophagy Flux and Lysosomal Biogenesis Depending on TFEB

**Figure 2:**
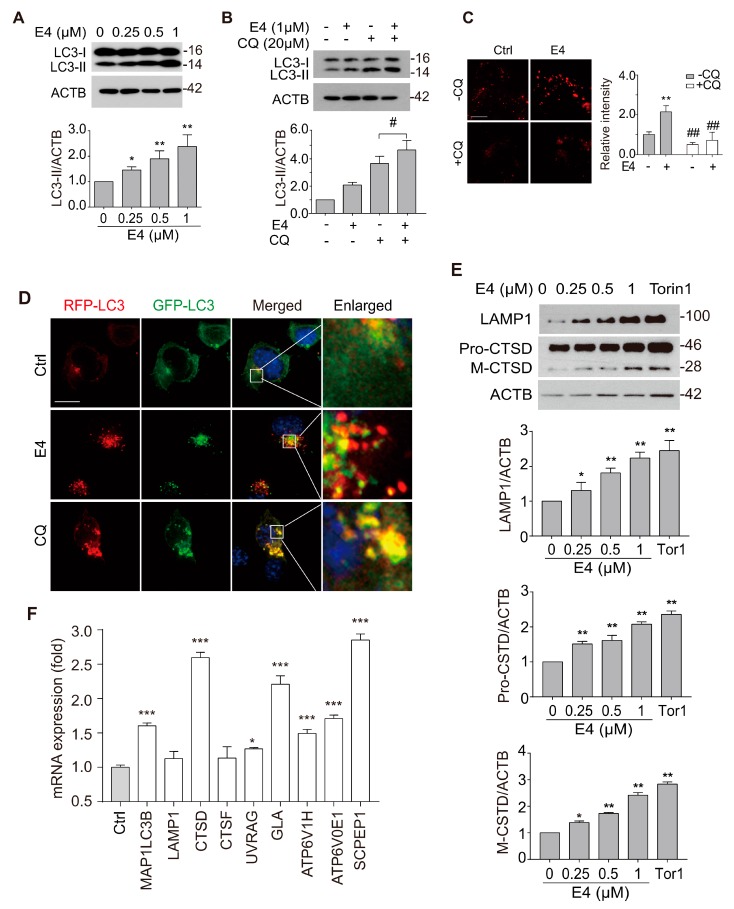
Compound E4 promoted autophagy flux and lysosomal biogenesis. (**A**) E4 dose-dependently increased the level of LC3B-II compared with vehicle control (0.1% DMSO). The expression of LC3-II in N2a cell after being treated with different concentration of E4 for 24 h were detected by Western blotting. Relative intensity of LC3B-II is normalized to that of β-actin/ACTB. Data are presented as mean ± SEM of three replicates in a representative experiment (**B**). The expression of LC3-II in N2a cell after being treated with indicated concentration of E4 and chloroquine (CQ) for 24 h were detected by Western blotting. Relative intensity of LC3B-II is normalized to that of β-actin/ACTB. Data are presented as mean ± SEM of 3 replicates in a representative experiment. (**C**) N2a cells were treated with vehicle control (0.1% DMSO), E4 (1 μM), or CQ (20 μM) for 12 h and then stained with LysoTracker Red DND-99 (50 nM) for 30 minutes. Fluorescence intensity of treated cells as measured by fluorescence microscopy. The numeric data are presented as means ± SEM from 3 independent experiments. (**D**) After the treated with vehicle control (0.1% DMSO), E4 (1 μM) or CQ (20 μM) in N2a cells that transfected with tf-LC3 plasmids for 16 h, the fluorescence signal was captured by fluorescence microscopy and representative images are shown. Scale bar: 15 μm. (**E**) E4 treatment increase LAMP1, CTSD levels in a dose-dependent manner as compared to vehicle control (0.1% DMSO). After N2a cells treated with E4 and positive control Torin1 (250 nM) at indicated concentration for 24 h. The expressions of LAMP1, pro-CTSD, mature-CTSD were detected by Western blot assay and quantified. (**F**) CF-7 cells were treated with E4 (1 μM) for 16 h. mRNA transcript abundance was assessed by real-time PCR using specific primers for the indicated genes. Relative quantification is presented as means ± SEM of 3 independent experiments. * *p* < 0.05, ** *p* <0.01, *** *p* < 0.001, ^##^ *p* < 0.01.

We next determined whether E4 enhances autophagy flux rather than causing lysosomal stress since lysosomal inhibition can activate TFEB [19]. First, we determined the effects of E4 on the autophagy maker LC3B. E4 dose-dependently increased the level of LC3B-II and the number of LC3B puncta determined by Western blot (Figure 2A) and immunostaining (Figure S2), respectively, in N2a cells, which indicated that E4 increases autophagosomes in cells. Secondly, in the presence of a lysosome inhibitor chloroquine (CQ) [20], E4 further increased the LC3B-II level (Figure 2B), indicating that E4 promotes the formation of autophagosomes. Thirdly, in N2a cells that were transfected with a tandem fluorescent mRFP-GFP-LC3 (tfLC3) plasmid [21], E4 treatment significantly increased the red-only puncta, whereas CQ treatment increased the yellow puncta (Figure 2D), thus further confirming that E4 promoted autophagy flux. For lysosome biogenesis, we found that E4 dramatically increased the lysosome contents, as determined by Lysotracker Red staining (Figure 2C), and increased the levels of LAMP1 and CTSD, as determined by Western blot (Figure 2E). At the gene level, E4 expectedly increased the mRNA levels of several autophagy and lysosome genes that were controlled by TFEB (Figure 2F).

**Figure 3:**
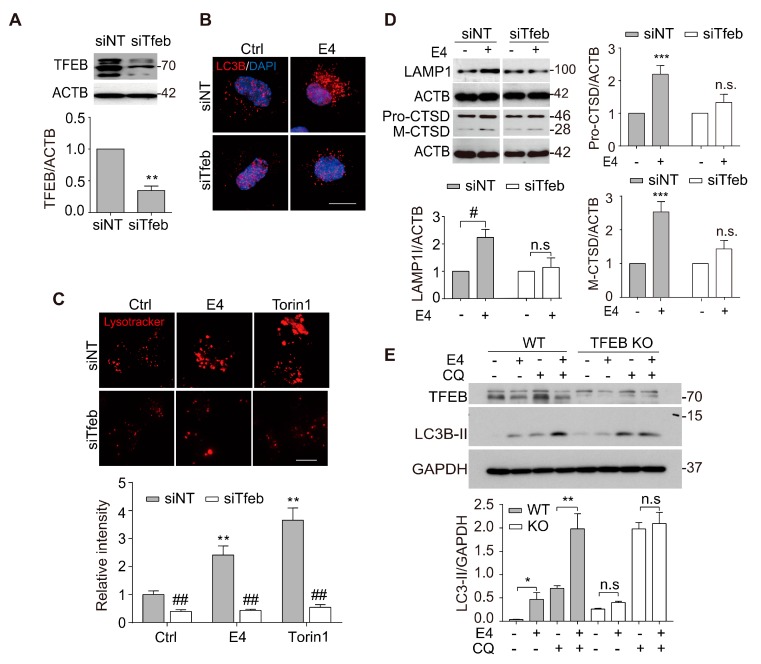
TFEB is required for E4-induced autophagy flux and lysosome biogenesis. (**A**) Transfected N2a cells with nontarget siRNA (*si-NT*, 50 nM) and *Tfeb* siRNA (*siTfeb*, 50 nm) for 48 h, and the expression of TFEB was evaluated by western blotting. Relative intensity of TFEB are normalized to that of ACTB/β-actin. The data are presented as the mean ± SEM from 3 independent experiments. (**B**) N2a cells were transfected with nontarget siRNA (*si-NT*, 50 nM), *Tfeb* siRNA (*siTfeb*, 50 nM) for 48 h and treated with E4 (1 μM) for 16 h. N2a cells treated with E4 (1 μM) and stained with anti-LC3 antibody. The fluorescence of LC3 puncta was detected by fluorescence microscopy, representative images are shown. Scale bar: 15 μm. (**C**) N2a cells were transfected with nontarget siRNA (*si-NT*, 50 nM), *Tfeb* siRNA (*siTfeb*, 50 nM) for 48 h and treated with E4 (1 μM) for 16 h and then stained with LysoTracker Red DND-99 (50 nM) for 30 minutes. Representative images are shown. Scale bar: 15 μm. Fluorescence intensity of treated cells as measured by fluorescence microscopy. The numeric data are presented as means ± SEM from three independent experiments. (**D**) N2a cells were transfected with nontarget siRNA (*si-NT,* 50 nM), *Tfeb* siRNA (*siTfeb*, 50 nM) for 48 h and treated with E4 (1 μM) for 16 h. The expressions of LAMP1 and CTSD were examined by Western blotting. Representative blots are shown. Relative intensity of LAMP1 and CTSD are normalized to that of ACTB/β-actin. Data are presented as the mean ± SEM from 3 independent experiments. (**E**) Wild type Hela cells and TFEB knockout cells were treated with E4 (1 μM) in the presence or absence of CQ (20 μM) for 16 h. Representative blots are shown. Relative intensity of LC3B-II is normalized to that of GAPDH. The data are presented as the mean ± SEM from three independent experiments. * *p* < 0.05, ** *p* < 0.01, *** *p* < 0.001, ^##^ *p* < 0.01, n.s. not significant.

We knocked down TFEB in N2a cells using *Tfeb* siRNA to determine whether TFEB activation contributes to the enhanced autophagy and lysosomal biogenesis induced by E4. Efficient knockdown (KD) of TFEB was confirmed by Western blot (Figure 3A). TFEB KD significantly abolished E4-induced increase in LC3B puncta and Lysotracker intensity (Figure 3B,C). Meanwhile, E4-induced increase in LAMP1 and CTSD was also significantly inhibited in the TFEB KD cells (Figure 3D). Furthermore, we confirmed the roles of TFEB in E4-induced autophagy in HeLa cells with TFEB knockout (KO). In wild-type (WT) cells, E4 significantly increased the level of LC3B-II in the presence or absence of CQ. However, no significant change in LC3B-II was observed in KO cells that were treated with E4 as compared to the control (Figure 3E). Together, our results confirmed that compound E4 promoted TFEB-mediated autophagy and lysosome biogenesis in vitro.

### 2.3. Compound E4 Activates TFEB Via Inhibiting AKT-MTORC1 Pathway

**Figure 4:**
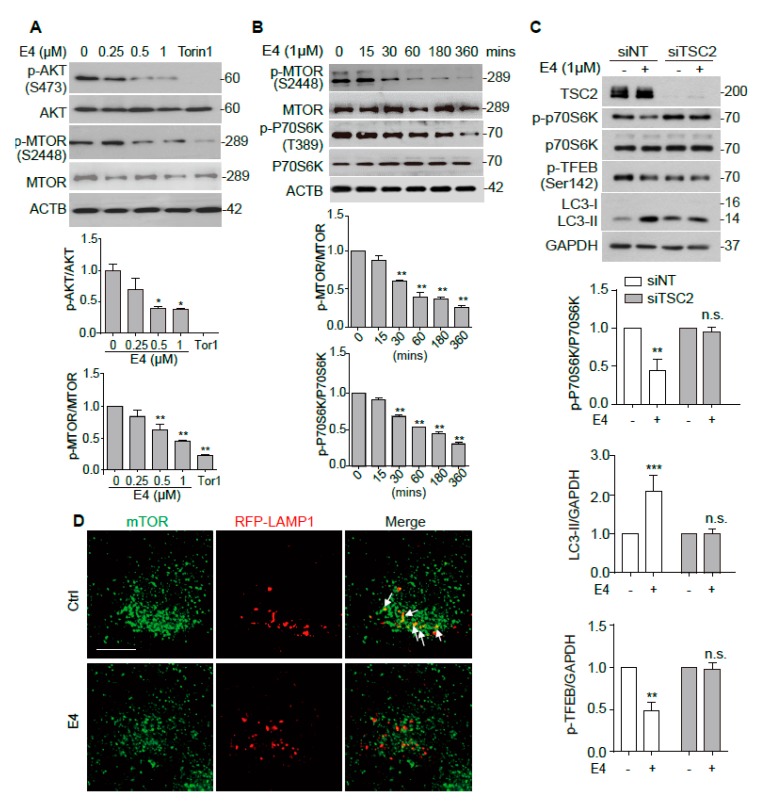
Compound E4 activates TFEB via inhibiting AKT-MTORC1 pathway. (**A**) N2a cells were treated with E4 at indicated concentration and positive control Torin1 (250 nM) for 6 h. Representative blots show the expression of phosphorylated (p-) and total AKT and mTOR. Data are presented as the mean ± SEM from three independent experiments. (**B**) N2a cells treated with 1 μM E4 for different durations (0–6 h). The expression of phosphorylated (p-) and total mTOR and p70S6K were examined by Western blotting. Data are presented as the mean ± SEM from three independent experiments. (**C**) CF-7 cells were transfected with nontarget siRNA (si-NT, 25 nM), TSC2 siRNA (si-TSC2, 25 nM) for 48 h and treated with E4 (1 μM) for 24 h. Representative blots show the expression of TSC2, phosphorylated (p-) and total mTOR, p-TFEB (Ser142) and LC3 proteins. The relative intensity of p-p70S6K is normalized to that of p70S6K. Relative intensity of p-TFEB and LC3-II is normalized to that of DAPDH. (**D**) N2a cells were transfected with mTOR and RFP-LAMP1 plasmids for 48 h and then treated with E4 (1 μM) for 24 h. The cells were stained with anti-mTOR antibody. Fluorescence was captured by fluorescence microscopy and representative images are shown. Scale bar: 25 μm. * *p* < 0.05, ** *p* < 0.01.

MTORC1 was shown to play a major role in the regulation of TFEB intracellular location [22]. MTORC1 is known to phosphorylate TFEB when nutrition is sufficient, and the inhibition of MTORC1 de-phosphorylates TFEB and promotes its nuclear translocation [23]. We examined the effects of E4 on the MTORC1 upstream kinase AKT, the phosphorylation of MTORC1, as well as substrate kinase P70S6K to determine whether E4 inhibits MTORC1 pathway to activate TFEB. E4 dose-dependently inhibited the phosphorylation of AKT and MTOR (Figure 4A). E4 also inhibited p-MTOR and p-P70S6K time-dependently (Figure 4B). Torin 1 was used as a positive control. We knocked down TSC2, a key upstream kinase of MTORC1, to continuously activate MTOR to further confirm the role of MTORC1 in E4-induced autophagy [24]. The results showed that E4 failed to inhibit p-P70S6K and increase LC3B-II in cells with TSC2 KD (Figure 4C). The phosphorylation of TFEB at Ser142 is controlled by MTORC1 [23]. E4 treatment reduced the level of p-TFEB (Ser142) in cells that were transfected with non-target (NT) siRNA, but not in cells transfected with TSC2 siRNA (Figure 4C). Furthermore, the colocalization of MTOR with LAMP1, which indicates the activation of MTORC1 [25], was abolished after E4 treatment (Figure 4D). Overall, these results suggest that E4 activates TFEB via inhibiting the AKT-MTORC1 pathway.

### 2.4. Compound E4 Reduces α-syn Levels and Protects Against MPP^+^ Toxicity in Vitro

**Figure 5:**
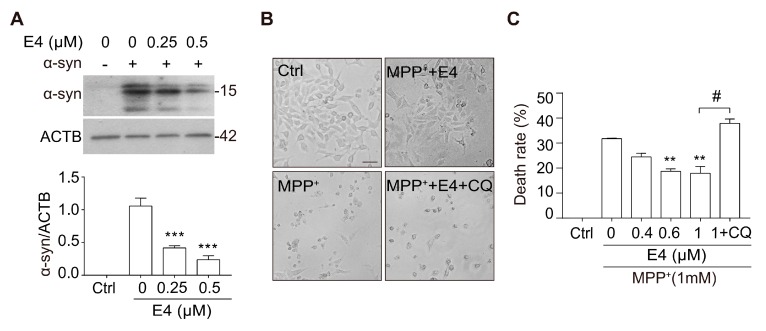
Compound E4 reduces α-syn levels and protects against MPP^+^ toxicity in vitro. (**A**) N2a cells were transfected with A53T α-syn plasmid and then treated with E4 at indicated concentration. Data are presented as the mean ± SEM from 3 independent experiments. (**B**) Bright field of PC12 cells after incubation with E4 (1 μM) present or absence of CQ (20 μM) in MPP^+^ (1 mM) treated cells. Scale bar: 50 μm. (**C**) Cell viability of E4 present or absence of CQ against MPP^+^ induced cell injury. Data are presented as the mean ± SEM from three independent experiments. * *p* < 0.05, ** *p* < 0.01, ^#^ *p* < 0.05, n.s. not significant.

TFEB activation has been shown to be protective in the cellular and animal models of PD [10,11,12,13,14]. Therefore, we primarily tested whether compound E4 exerts neuroprotection in cellular models of PD. In N2a cells transfected with A53T α-syn plasmid, E4 treatment dose-dependently reduced the level of overexpressed α-syn (Figure 5A). The MPP^+^ (1-methyl-4-phenylpyridinium ion) is the most common neurotoxin that is used for modeling PD, since it mimics the slow neuronal cell death of PD [26]. We pre-treated PC12 cells with E4 for six hours and then co-treated cells with MPP^+^ for 48 h. The cell viability results showed that E4 significantly reduced cell death and dose-dependently protected PC12 cells against MPP^+^-induced cytotoxicity (Figure 5B,C). Cell viability was measured by Alamar blue assay. Interestingly, this neuroprotective effect was abolished by co-treating cells with lysosome inhibitor CQ, suggesting that the neuroprotective effects of E4 are through the autophagy-lysosome pathway (Figure 5B,C). Taken together, E4 has shown good neuroprotective effects in two different PD cell models.

## 3. Discussion

**Figure 6:**
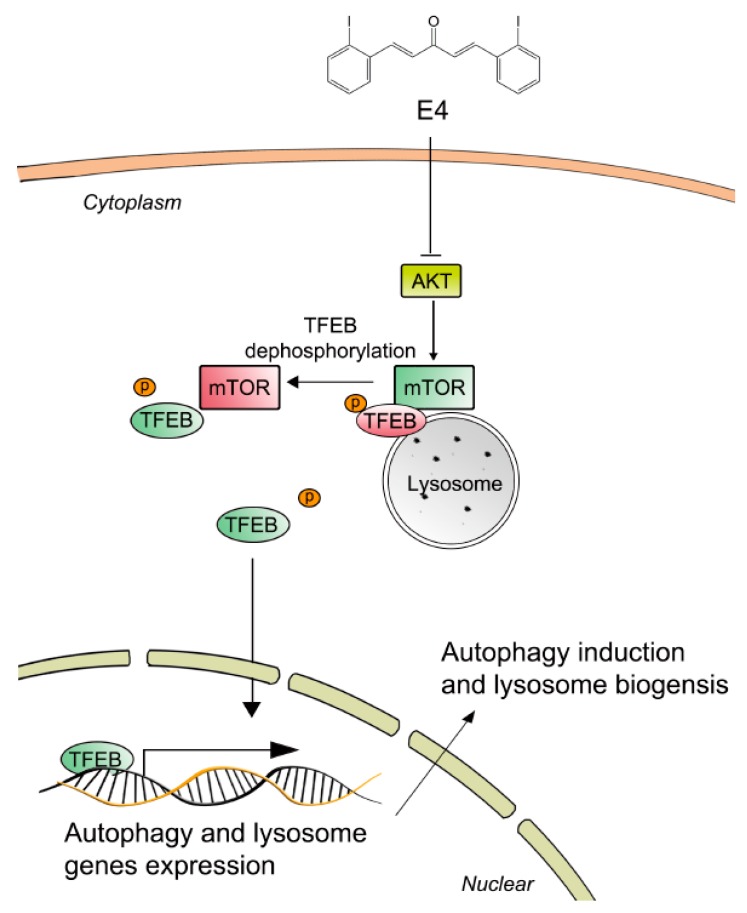
Underlying mechanisms by which compound E4 activate TFEB and autophagy-lysosome process. Compound E4 inhibited AKT as well as MTORC1 pathway and further promoted the nuclear accumulation of TFEB. Cytoplasmic TFEB is highly phosphorylated and located in the surface of lysosome which bind with MTOR. Compound E4 inhibited the MTOC1 activity and promoted TFEB dephosphorylation. Dephosphorylated TFEB translocated into nucleus and bind to autophagy and lysosome related genes site. Eventually, compound E4 promoted TFEB from cytoplasm to nucleus and induced autophagy and lysosome biogenesis.

In this study, we discovered a novel TFEB activator, named E4, from synthesized curcumin derivatives. By activating TFEB, E4 promoted autophagy flux and lysosomal biogenesis, which are essential for the clearance of protein aggregates, as seen in neurodegenerative diseases, including PD (Figure 6).

The activation of TFEB is mainly controlled by its phosphorylation. TFEB is phosphorylated by several regulators, such as GSK3B, AKT, MTORC1, and ERK2, and it remains inactive in the cytoplasm [18]. MTORC1 is one of the most important regulators for TFEB by controlling its phosphorylation [27]. The phosphorylation site of Ser142 is regarded as an essential site for MTORC1 to regulate TFEB at the surface of lysosome membrane. So far, our data revealed that AKT-MTORC1 is involved in E4-induced TFEB activation. However, whether E4 acts on other upstreaming regulators of TFEB needs to be fully evaluated.

To date, there is no cure for PD. New pathological mechanisms and new drug targets are extensively explored. The impairment of ALP in PD has been increasingly recognized and new drugs promoting ALP are being discovered and evaluated in PD models [6,7]. Since TFEB controls the whole ALP process, it presents an ideal target for PD drug development [12]. Major cell models of PD are α-syn overexpression model and the neurotoxins induced cytotoxicity model. Recent studies indicated that MPP^+^ impaired the autophagy flux and induced α-syn aggregation [28,29,30]. Additionally, MPP^+^ treatment was also found to decrease the LAMP1 level in human dopaminergic BE-M17 neuroblastoma cells, as well as MPTP, treated mice brain [31]. Consequently, the MPP^+^ cell model might be an appropriate model for studying the protection of PD. The aggregation of α-syn has been reported to block autophagy flux, whereas enhanced autophagy might promote α-syn degradation [28,32]. Our preliminary data showed that the protective effects of E4 in these cell models. However, whether E4 has good brain bioavailability and, therefore, ideal neuroprotective efficacy in PD animal models needs to be further investigated.

## 4. Material and Methods

### 4.1. Materials

The analog of curcumin E4 was synthesized according to our previous study and stored in DMSO (Stock concentration 1 mM) at −35 °C. MPP^+^ (1-Methyl-4-phenylpyridinium iodide) (D048) and anti-Flag (F1804) antibody were purchased from Sigma-Aldrich. (St. Louis, MO, USA) TFEB siRNA (L-050607-02-0005), TSC2 siRNA (L-003029-00-0005), and non-target siRNA were purchased from Dharmacon. Torin1 (2273-5) was purchased from BioVision Inc. Anti-β-actin/ACTB (sc-488 47778) and anti-α-synuclein (c20) (J1615) were purchased from Santa Cruz Biotechnology. Anti-LAMP1 (AB24170) and anti-CSTD (AB75852) antibody were purchased from Abcam Company (Abcam, Cambridge, UK). Anti-phospho-P70S6K (Thr389) (9234), anti-P70S6K/RPS6KB1 (9202), anti- H3F3A/histone H3 (D1H2), anti-phospho-mTOR (Ser2448) (2971S), anti-mTOR (2972S), anti-α-synuclein (2628S), anti-TSC2 (4308s), anti-AKT (9272S), and anti- phosphor-AKT (Ser473) (9276S) antibodies were purchased from Cell Signaling Technology company. Anit-TFEB (A301-852A) antibody was purchased from Bethyl Laboratories, Inc. Anti-TFEB (Ser142) (Q3004775) was purchased from Millipore Company. Anti-GAPDH (GTX100118) antibody was purchased from GeneTex Company. Anti- LC3B (NB100-2220) was purchased from Novus Biologicals. LysoTracker® Red DND-99 (L-7528), DMEM (11965-126), FBS (10270-106), Opti-MEM I (31985-070), and alamarBlue™ Cell Viability Reagent (DAL1025) were purchased from Life Technologies. TRIzol™ Reagent (15596018) was purchased from Thermo Fisher Scientific Inc. PrimeScript™ RT Master Mix (Perfect Real Time) (RR036A) was purchased from Takara Bio Company. Alexa Fluor®488 goat anti-mouse IgG (A-11001), Alexa Fluor®488 goat anti-Rabbit IgG (A-11034), and Alexa Fluor 488 (A-11008) were purchased from Life Technologies (Carlsbad, CA, USA).

### 4.2. Cell Culture and Drug Treatment

N2a cells, PC12 cells, wild type Hela cells, and TFEB knock out Hela cells were cultured in DMEM supplemented with 10% FBS. Wild type Hela cells and TFEB knock out Hela cells were the gift from Prof. Richard J. Youle (National Institute of Neurological Disorders and Stroke) and Prof. Myung-Shik Lee (Yonsei University College of Medicine). The HeLa cells stably expressing 3x-Flag-TFEB (CF-7) cells were maintained in DMEM that was supplemented with 10% FBS and 50 μg/mL G418 [27]. All of the cells were maintained with cell culture medium containing 100 units/mL penicillin/streptomycin mixture (Invitrogen, Carlsbad, CA, USA) at 37 °C, gassed with 5% CO_2_. For drug treatment, the full medium was replaced by fresh Opti-MEM I containing the compounds (in 0.1% DMSO) and then incubated for the indicated time periods.

### 4.3. Cell Transfection and Gene Knockdown Assay

For overexpression, the cells were transfected by using Lipofectamine 3000 (L3000015) following the manufacturer’s instruction. For gene knockdown, the cells were transfected with siTFEB or siTSC2 and the nontarget siRNA by using Lipofectamine RNAiMAX (Invitrogen, 13778030) and incubated at 37 °C for 48 h. The cells were treated with indicated drugs after being transfected with siRNAs and plasmids for 48–72 h.

### 4.4. Quantitative Real-Time PCR

Total RNA was extracted from cells while using the TRIzol™ Reagent (15596018). Reverse transcription was performed using PrimeScript™ RT Reagent Kit with gDNA Eraser (Perfect Real Time) (Takara Bio Inc, Shiga, Japan, RR047A). Autophagy and lysosome gene primers were retrieved from Origene and from a previous study [10] and synthesized by Life Technologies. Table S1 lists the oligonucleotide sequences. Real-time PCR was carried out with the PrimeScript™ RT Master Mix (Perfect Real Time) (RR036A) using the ViiA™ 7 Real-Time PCR System (Life Technologies, Carlsbad, CA, USA). Fold changes were calculated while using the ∆∆CT method, and the results were normalized against an internal control (GAPDH).

### 4.5. Alamar Blue Assay

Add 1 of 10 volume of alamarBlue™ Cell Viability Reagent (DAL1025, Life Technologies) directly to cells in the culture medium, incubate for one to four hours at 37 °C in a cell culture incubator, being protected from direct light. Monitor the absorbance of Alamar Blue at 570 nm, using 600 nm as a reference wavelength. Results of treatment were read relative to controls, while assuming the absorbance of controls was 100%.

### 4.6. Isolation of the Cytosol and the Nucleus Fractions

Cytosol and nucleus extracts were prepared according to a previous protocol. In brief, at the end of drug treatment, the cells were washed with ice-cold PBS, centrifuged, and then re-suspended in cold lysis buffer containing 20 mM N-2-hydroxyethylpiperazine-N’-2-ethanesulfonic acid (HEPES), pH 8.0, 1 mM ethylenediaminetetraacetic acid (EDTA), 1.5 mM MgCl2, 10 mM KCl, 1 mM DTT, 1 mM sodium orthovanadate, 1 mM NaF, 1 mM PMSF, 0.5 mg/mL benzamidine, 0.1 mg/mL leupeptin, and 1.2 mg/mL aprotinin. The cells were allowed to swell on ice for 15 min. NP-40 (10% (v/v)) was subsequently added to the cell suspensions. The samples were vigorously vortexed for 10 s. The homogenates were centrifuged for 50 s at 16,000× *g*, and the supernatant was used as cytosolic extract. The nuclear pellet was re-suspended in cold extraction buffer containing 20 mM HEPES, pH 8.0, 1 mM EDTA, 1.5 mM MgCl2, 10 mM KCl, 1 mM DTT, 1 mM sodium orthovanadate, 1 mM NaF, 1 mM PMSF, 0.5 mg/mL benzamidine, 0.1 mg/mL leupeptin, 1.2 mg/mL aprotinin, and 20% glycerol. All of the protein fractions were stored at −30 °C until use.

### 4.7. Western Blot

The cells were washed twice with cold 1x PBS (phosphate-buffered saline) and then lysed on ice with 1× RIPA Lysis buffer (9803, Life Technologies) with complete protease inhibitor mixture (04693124001, Roche Applied Science, Penzberg, Germany) and phosphatase inhibitor (B15001, Bio-connect BV, Huissen, Netherlands). The protein concentrations were determined by Bio-Rad Bradford assays while using bovine serum albumin (BSA) as standard. Proteins were separated by 10–15% SDS-PAGE, transferred, and blotted with the antibodies described. The blots were then incubated with primary and secondary antibodies. The protein signals were detected by the ECL kit (Pierce, 32106) and quantified while using ImageJ software (Version 1.52, NIH, USA).

### 4.8. Immunocytochemistry

The cells were seeded on coverslips placed in 24-well plates. For autophagy flux assessment, N2a cells were transfected with tfLC3 plasmid for 24 h and then treated with the indicated drugs. For lysosome activity was evaluated using LysoTracker Red DND99 (L7528, Thermo Fisher Scientific, Massachusetts, USA) according to the manufacturer’s instructions. During the last 1 h of drug treatment, 50nM LysoTracker Red DND99 added to plates. After treatment, the slices were washed three times with PBS and fixed with 4% paraformaldehyde, permeabilized in 0.25% Triton X-100 (Sigma-Aldrich, St. Louis, MO, USA, T8787) and blocked with 3% BSA. After blocking, slides were stained with anti-Flag (1:600), anti-TFEB (1:200), and anti-LC3B (1:500) antibodies overnight at 4 °C. Alexa Fluor® 488 (green) secondary antibodies (1:1000) were added for 1 h at room temperature. After nuclear staining with DAPI, the slices were mounted with FluorSave reagent (Calbiochem, Darmstadt, Germany, 345789). The cells were visualized while using the API DeltaVision Personal Imaging System and Confocal Laser Scanning Microscope (Leica TCS SP8, Wetzlar, Germany).

### 4.9. Statistical Analysis

Each experiment was performed at least three times, and the results were presented as mean ± SEM. Student’s t-test or One-way analysis of variance (ANOVA), followed by the Student-Newman-Keuls test while using GraphPad Prism 7.0 software (San Diego, CA, USA) packages. A probability value of *p* < 0.05 was considered to be statistically significant.

## Supplementary Materials

Supplementary materials can be found at https://www.mdpi.com/1422-0067/21/4/1515/s1.
