# Supplementary figures and images for: A Curcumin Derivative Activates TFEB and Protects Against Parkinsonian Neurotoxicity in Vitro
Source: Int J Mol Sci. 2020 Feb 22;21(4):1515. doi: 10.3390/ijms21041515 (PMC7073207; doi:10.3390/ijms21041515)

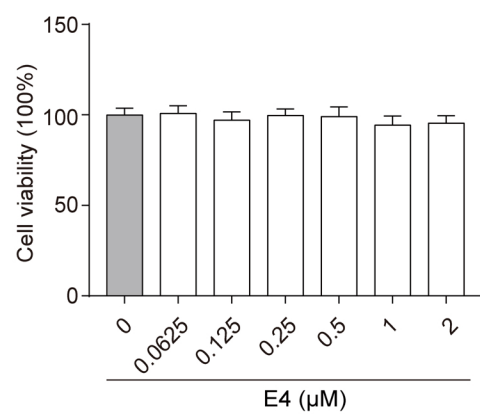

**Figure S1**

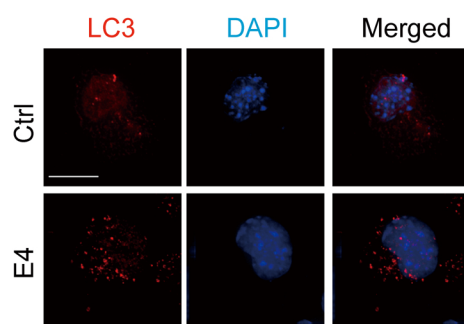

**Figure S2**

Supplement: Supplementary file 1 [file ijms-21-01515-s001.pdf]
